# Characterizing the Impact of the COVID-19 Pandemic on HIV PrEP care: A Review and Synthesis of the Literature

**DOI:** 10.1007/s10461-022-03941-w

**Published:** 2022-12-02

**Authors:** Chenglin Hong

**Affiliations:** Department of Social Welfare, UCLA Luskin School of Public Affairs, 3250 Public Affairs Building, 90095-1656 Los Angeles, CA USA

**Keywords:** pre-exposure prophylaxis, PrEP, COVID-19, HIV/AIDS, review

## Abstract

The global COVID-19 pandemic and associated lockdown measures have caused disruptions to sexual health services and created additional barriers to the continuity of HIV pre-exposure prophylaxis (PrEP) among key populations. This review provides an examination of the influences of the pandemic on engagement in the PrEP care continuum. Using the PRISMA guideline, 46 studies were included in this review and the synthesis. Most of the studies were conducted in high-income settings through quantitative analysis. A majority of studies examining the changes in PrEP use suggested a decline or discontinuation in PrEP uptake during the pandemic. The most common reasons for stopping using PrEP were perceived barriers to PrEP-related care, having reduced sexual behaviors and fewer sexual partners, and reduced perceived risk of HIV infection. Limited studies documenting an increase in PrEP uptake were all in specific PrEP optimizing programs. During the pandemic, there is also an emerging trend of switching to on-demand PrEP from daily oral PrEP. Future studies should understand the mechanism of strategies that facilitated the improvements during the pandemic. PrEP implementation programs should consider alternative PrEP modalities and provide consistent and comprehensive knowledge about correct information.

## Introduction

The COVID-19 pandemic and its associated measures have posed significant challenges to health systems and access to healthcare services across the globe. These unprecedented consequences may increase health inequality and exacerbate the disparities that already exist among the most vulnerable populations, such as people living with and are at risk of HIV infection. Since the beginning of the COVID-19 crisis, there has accelerating evidence suggesting that the ongoing pandemic has changed the provision of sexual health services and created additional social and structural barriers to HIV prevention services[1]. These consequences could not only threaten the public health gains in HIV prevention in the past decade but also severely impact the overall progress of the HIV/AIDS 90-90-90 targets[2, 3]. For example, in a recent report published by the United States (U.S.) Centers for Disease Control and Prevention (CDC), HIV testing had dropped dramatically in both healthcare and non-healthcare settings during the first year of the pandemic[4], particularly among priority populations such as men who have sex with men (MSM) and transgender individuals. Similarly, data from 65 primary care clinics in South Africa showed a nearly 50% decrease in HIV testing at the beginning of the lockdown measures[5]. Results from multiple mathematical models also suggested that the potential COVID-19 related effects of disruptions to the HIV program could lead to increased HIV incidence and population-level mortality[6]. Urgent programmatic and policy responses are needed to mitigate the negative effects of the COVID-pandemic on the existing HIV epidemic.

One key strategy to prevent HIV transmission and End the HIV Epidemic is to scale up and implement pre-exposure prophylaxis (PrEP) among the key community and populations[7]. PrEP is a medicine taken to prevent getting HIV infection and reduce the risk of getting HIV when taken as prescribed[8]. Both clinical trials and real-world longitudinal studies have shown the effectiveness of PrEP in reducing HIV incidence among populations at elevated HIV risk[9–14]. Previous modeling work has also demonstrated the potential benefit of scaling-up PrEP and expanding PrEP coverage in averting HIV infections across different countries and settings[15–19]. Until the 1st quarter of 2022, it is estimated that nearly 2.3 million individuals had initiated PrEP worldwide[20]. Undoubtedly, the pandemic and associated measures also disrupted the continuity of PrEP care across the globe among priority populations. For example, in the U.S., one in 7 young MSM PrEP users stopped using it during the pandemic[21]. In Wales, the introduction of COVID-19 related measures was associated with reducing PrEP use and PrEP adherence[22], and similar phenomena were reported in many other settings[23–26]. The report from U.S. CDC also suggests a slowing PrEP prescription in 2020. However, these findings were not consistent. In fact, one study in South Africa observed more PrEP initiation, improved persistence, and lower risks of client-initiated stops among female sex workers in 2020, compared to the pre-pandemic period[27].

Importantly, ten years after oral PrEP was approved for HIV prevention, there is still a critical gap in implementing this biomedical intervention, including lower utilization among key populations and suboptimal PrEP adherence around the globe. The ongoing pandemic and associated measures may pose increased HIV risk and additional barriers to the broader use of PrEP among those in need. Understanding these barriers, particularly during the COVID-19 pandemic, and examining the changes in PrEP use are paramount to ensuring its effective implementation and public health benefit. Therefore, the objectives of this review are (1) collectively describe the changes in PrEP use and persistence during the pandemic; (2) to summarize the factors associated with and reasons for these changes, and (3) to describe the emerging PrEP modalities during the COVID-19 pandemic. The results of this review will provide experiences and lessons we learned from the pandemic, which could support the optimization of future programs and policy changes, especially for the preparation of future endemics and pandemics that might reduce the delivery and use of sexual health care services.

## Methods

This review was conducted in line with the Preferred Reporting Items for Systematic Review and Meta-Analysis (PRISMA) guideline[28]. A comprehensive database search was performed in the following databases in July 2022: PubMed, Embase, PsycInfo, CINAHL, Web of Science, and Medline for articles published since 2020. The search terms include (*COVID-19 OR SARS-CoV-2 OR Severe acute respiratory syndrome coronavirus 2 OR 2019nCoV OR HCoV-19*) AND (*Pre-exposure prophylaxis OR PrEP OR pre-exposure chemoprophylaxis OR Truvada OR Descovy*). A manual search was also conducted for other relevant studies using reference lists of retrieved citations and Google Scholar. Additionally, conference abstracts for major HIV conferences (AIDS 2020, CROI 2021, Adherence 2021) were searched to identify additional relevant records.

To be eligible for inclusion, studies had to be (1) in the English language or have an English Abstract; (2) study data were collected since March 2020, when the World Health Organization declared a global pandemic or associated lock-down measures were implemented in the study setting; (3) have at least one outcome of PrEP use and/or (dis)continuation in their study findings. To characterize the impact of COVID-19, studies that described the changes or disruptions in the delivery of PrEP were also included. Besides, all types of studies were included if they provided empirical data.

All database search records were imported to Covidence (Veritas Health Innovation Ltd), a software that removes duplicate studies automatically. After duplicates were removed, all article titles and abstracts were screened for eligibility, and if they met the inclusion criteria, the study was included for full-text review. Any studies that cannot be decided solely by title and abstract were also progressed to full-text review. Next step, all articles were screened to determine whether they met all the eligibility, and those that met all the criteria were included in the final data extraction. For each selected article, standard data (author, year published, study design, study population) and study-specific information (PrEP use and (dis)continuation, changes in PrEP delivery, behaviors among PrEP users, etc.) were imported into a standardized data extraction form. Descriptive statistics were used to present the characteristics of included studies, including study location, population, and study design. Inductive thematic analysis was used to qualitatively identify themes across article findings, which are reported by overarching themes along with supporting evidence and quotes extracted from included articles.

## Results

### Study Selection

**Fig. 1:**
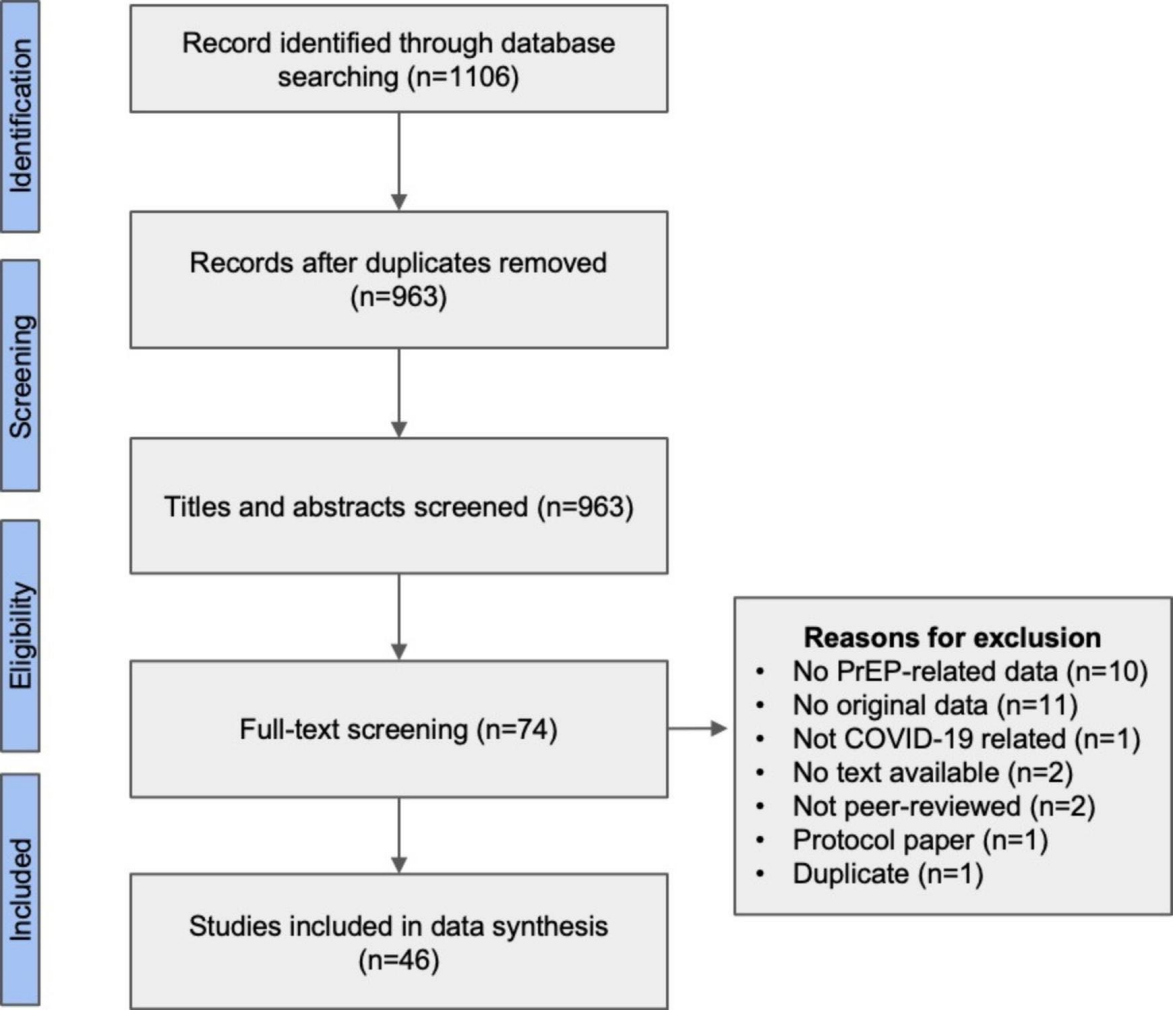
PRISMA flow diagram for study selection: the impact of the COVID-19 pandemic on HIV PrEP care

The initial database search yielded 1106 records. After removing duplicates, 963 studies were processed for the title and abstract review. Among those, 74 records met the eligibility and therefore were included for full-text review. After reviewing the full article, 46 studies met the inclusion criteria and were included in the final data extract and synthesis. Study selection was presented in a modified PRISMA flowchart (see Fig. 1).

### Characteristics of Included Studies

A full description and details of included studies’ characteristics are summarized (see Table 1**)**. These studies were conducted in the United States (*n* = 18)[21, 29–45], United Kingdom (*n* = 6)[22, 46–50], Australia (*n* = 4)[23, 51–53], Brazil (*n* = 4)[54–57], Portugal (*n* = 3)[54, 56, 58], Kenya (*n* = 2)[59, 60], Belgium (*n* = 2)[24, 26], South Africa (*n* = 2)[25, 27]. The rest of the studies were conducted in Canada[61], Croatia[62], Ireland[63], Zimbabwe[64], Netherlands[65], the Philippines[66], France[67]. One study included data from 20 different countries[68]. Most studies (n = 42) utilized quantitative methods, with 2 qualitative studies[29, 43] and 2 mixed-method designs [39, 60]. A majority of studies (n = 27) were comprised of MSM, three of which explicitly focused on young MSM[21, 29, 30]. Four studies included transgender women, two among female sex workers[27, 64], one study was conducted among people who inject drugs (opioid-dependent individuals)[38], and one among pregnant women[25].

**Table 1:** Characteristics of studies examining the impact of the COVID-19 pandemic on HIV PrEP care (*n* = 46)

Characteristics	*n* (%)
Study location*	
United States	18
United Kingdom	6
Australia	4
Brazil	4
Portugal	3
Kenya	2
Belgium	2
South Africa	2
Canada	1
Croatia	1
Ireland	1
Zimbabwe	1
Netherlands	1
Philippines	1
France	1
Global sample	1
*Study design*	
Quantitative	42 (91.3%)
Qualitative	2 (4.3%)
Mixed methods	2 (4.3%)
Study population*	
men who have sex with men	27
Transgender women	4
Female sex workers	2
Pregnant women	1
People who inject drugs	1

*Studies with multiple locations/population prevent assessment of proportion of study locations

### Changes in PrEP use and Continuation

Many studies documented the decline or discontinuation of PrEP uptake among individuals who used PrEP pre-pandemic worldwide. Chan et al. found that 12% of individuals discontinued PrEP after the shelter-in-place order was initiated in San Francisco[45]. In a national cohort of cisgender men, transgender women, and transgender men who have sex with men in the U.S., nearly 30% of the sample completed stopped taking PrEP[41]. Studies in Australia and France noted even higher rates of PrEP discontinuation during the pandemic, with 41.8% and 58.8% of MSM stopping PrEP during the lockdown and COVID-19-related restrictions[52, 67]. In Amsterdam, Netherlands, Jongen et al. suggested that among MSM reported study data before and after March 15th 2020, two in three (67%) decreased PrEP uptake after March 15th, 2020[65]. Similarly, researchers in Wales also found that the odds of participants taking PrEP on a given day were 56% lower after introducing COVID-19 measures[22]. These changes have been noted among several populations at high risk for HIV infection. Among a national sample of YMSM aged 17–24 years old in the U.S., Hong et al. found that 1 in 7 YMSM stopped using PrEP during the pandemic[21]. Two studies conducted in Australia and France also discovered that compared to those who continued using PrEP, those who had discontinued PrEP during the COVID-19 restrictions were younger[52, 67].

Conversely, one study in San Francisco suggested that, based on their data from the San Francisco primary care clinics, there was no reduction in PrEP use after the shelter-in-place orders were implemented[33]. Three other studies found that PrEP uptake had increased since the pandemic began. According to Muhula et al. (2021), PrEP uptake had increased by 24% among the various population in Kibera, Kenya, adjusting for other study variables[59]. Matambanadzo et al. (2021) discovered that among female sex workers in Zimbabwe, PrEP uptake has increased monthly since May 2020 and peaked at an initiation rate in September 2020[64]. Lastly, among female sex workers at TB HIV care in eThekwini, South Africa, researchers observed more PrEP initiation, improved PrEP persistence, and lower discontinuation during the pandemic[27] (see Table 2).

**Table 2:** Summary of findings in studies examining the impact of the COVID-19 pandemic on HIV PrEP care

Author (year)	Country/setting	Study design	Study population	Sample size	Summary of findings
Bellman (2022)	San Francisco, USA	Qualitative	n/a	n/a	COVID-19 delayed implementation of SB-159 because of an increase in demand for COVID-19 services at pharmacies (testing and vaccinations), which reduced the resources and number of staff available. Some pharmacies noted a reduced number of requests for PrEP and PEP services, which they believed was due to the effects of social distancing and decreased sexual activity during the pandemic.
Bogdanić (2020)	Croatia	Quantitative	n/a	n/a	PrEP service had a 50% decline in visits (12 versus 6 visits per week in February and March 2020, respectively).
Camp (2021)	USA	Quantitative	MSM	n = 140	The most common reasons for switching from once daily to 2-1-1 PrEP included having sex less frequently and wanting to take fewer pills (46.4%). Participants reported high medication adherence based on each component of 2-1-1 PrEP dosing (> 84%). The most common barriers with 2-1-1 PrEP dosing included unplanned sexual encounters resulting in missing the double-dose pre-sex and trouble remembering doses post-sex. Facilitators of the 2-1-1 PrEP dosing strategy included reductions in sexual encounters, preference to take fewer pills, need to reduce cost, and desire to reduce side effects. Challenges to receiving PrEP services during the pandemic included obtaining laboratory testing and PrEP refills (either receipt of a refill authorization from a healthcare provider or processing of a refill from the pharmacy), being unable to get a healthcare provider appointment, and not being able to communicate with their healthcare provider.
Chan (2022)	San Francisco, USA	Quantitative	n/a	n = 74	67% of participants were taking daily PrEP, 21% were on 2-1-1 PrEP, and 12% had discontinued PrEP. 53% of participants reported challenges with taking PrEP. The most reported challenges were related to health care system access, including inability to go to laboratories for testing, inability to receive PrEP refills, lack of communication with their health care providers, and/or clinics not having available appointments. In addition, 51% of participants noted at least some difficulty getting an HIV test. Nearly 30% of participants were uncertain about taking PrEP while having less sex, with the number of sexual partners significantly reduced from a mean of 4.0 sexual partners per month before SIP to a mean of 2.3 during shelter in place (P < 0.01).
Chen (2021)	Chicago, USA	Quantitative	Black MSM and TW	n = 222	Most (83.3% and 78.2%, respectively) reported similar or easier PrEP access during the pandemic. After adjusting for covariates, financial travel burden since the shelter-in-place was significantly associated with perceived difficulty in accessing PrEP [aPR = 3.2 (95% CI: 1.0 to 10.1)]
Chone (2021)	Portugal	Quantitative	MSM	n = 1301	30.6% of participants were using PrEP. Using PrEP was associated with having chemsex during the pandemic (ORa: 4.2, 95%CI 2.71–6.39)
Chow (2020)	Melbourne, Australia	Quantitative	MSM	n = 204	One in 4 daily PrEP users stopped taking PrEP during the COVID-19 pandemic, and 5% switched to on-demand PrEP. Most men reduced PrEP use because they stopped having casual sex and reduced the number of casual partners during the COVID-19 pandemic.
Chow (2020)	Melbourne, Australia	Quantitative	MSM	n = 192	80% PrEP users did not change how they took PrEP. Of the 136 daily PrEP users, 111 (82%) continued to take daily PrEP, 3 (2%) switched to on- demand PrEP, and 22 (16%) stopped PrEP in July–August. Men generally reported that they had no partners or decreased sexual activities during second lockdown compared with post-first lock- down; the number of casual sex partners (43% decreased vs. 3% increased) and the number of kissing partners (36% decreased vs. 3% increased).
Davey (2020)	South Africa	Quantitative	Pregnant women	n = 455	During the nationwide lockdown, missed PrEP visits increased significantly to 63% at the 1-month visit and 55% at the 3-month visit. Overall, 34% of women missed visits before lockdown and 57% during lockdown. The relative risk of missing a study visit increased during lockdown compared with before lockdown (odds ratio 2∙36, 95% CI 1∙73–3∙16).
De Daetselier (2021)	Belgium	Quantitative	n/a	n/a	Approximately the same number of PrEP users was seen in 2020 (n = 905) compared to 2019 (n = 912). However, participants visited the clinic less frequently in 2020 as compared to 2019. For example, the number of PrEP users that visited the clinic more than twice was only half of the number observed in 2020 (17.1% (155/905) versus 34.2% (312/912) in 2020).
De Sousa (2021)	Brazil/Portugal	Quantitative	MSM	n = 2361	Notably, among the 652 users of PrEP/Truvada in this study, almost half (301; 46.1%) also stated using this medicine as a preventive measure to COVID-19 transmission.
Di Ciaccio (2021)	France	Quantitative	MSM	n = 8345	Among 8345 respondents, 946 were PrEP users before the lockdown, of whom 58.8% (n = 556) reported stopping PrEP during the lockdown and 15.4% (n = 146) were not using PrEP at the time of the survey. Among the 556 who stopped PrEP during lockdown, 86.5% (n = 481) reported no sexual activity; 76.8% (n = 427) restarted PrEP after lockdown. Former PrEP users were more likely to be younger, not living with a stable male sexual partner, report moderate anxiety, report increased psychoactive drug use during the lockdown, and report not having tested for HIV or STI since the end of the lockdown because they did not know where to go, preferred to wait or for another reason. Reporting fewer male sexual partners in the last 6 months was also significantly associated with being a former PrEP user.
Eustaquio (2022)	Western Visayas, Philippines	Quantitative	Cis-MSM and TGW	n = 647	Among those non-reactive for HIV testing, all participants were provided prevention services through routine provision of risk reduction counseling and condoms and lubricants. But only 2 (0.3%) were successfully linked to PrEP services.
Gillespie (2020)	Wales	Quantitative	n/a	n = 56	On average, 42% of PrEP users reported condomless sex in the period prior to the introduction of social distancing measures and 20% reported condomless sex after (OR = 0.16, 95% CI 0.07 to 0.37, p < 0.001). There was some evidence to suggest that this association was moderated by relationship status (OR for single participants = 0.09, 95% CI 0.06 to 0.23; OR for not single participants = 0.46, 95% CI 0.16 to 1.25).
Gillespie (2022)	Wales	Quantitative	n/a	n/a	Prior to the introduction of control measures, PrEP was taken on 3791/5785 (66%) days, there were CAS episodes on 506/5559 (9%) days, and 207/406 (51%) of CAS episodes were covered by an adequate amount of daily PrEP. The introduction of pandemic-related control measures was associated with a reduction in PrEP use (OR 0.44, 95%CI 0.20–0.95), CAS (OR 0.35, 95%CI 0.17–0.69), and PrEP adherence (RR = 0.55, 95%CI 0.34–0.89). The odds of participants taking PrEP on a given day were 56% lower following the introduction of control measures (OR 0.44, 95% CI 0.20 to 0.95, z = − 2.09, p = 0.037).
Grov (2021)	USA	Quantitative	TGW and MSM	n = 789	Among the 789 participants prescribed HIV pre-exposure prophylaxis (PrEP), 29.9% said they stopped taking their PrEP entirely, and 14.2% started selectively skipping doses. For those who had been taking PrEP, discontinuing PrEP was associated with having no new sex partners (β = 0.90, 95% CI 0.40–1.40).
Hammoud (2021)	Australia	Quantitative	MSM	n = 847	41.8% (n = 167) discontinued PrEP use during COVID-19 restrictions. Discontinuing PrEP during COVID-19 restrictions was independently associated with being less likely to have recently tested for HIV (aOR: 0.17; 95% CI: 0.09 to 0.34; P < 0.001) and less likely to report sex with casual partners (aOR: 0.28; 95% CI: 0.14 to 0.54; P < 0.001). Among the 167 men who discontinued using PrEP during COVID-19 restrictions, the majority reported sex with casual partners in the 6 months before survey completion between 2015 (78.3%) and 2019 (69.8%), but this fell to 7.8% during COVID-19 restrictions. Men who discontinued PrEP during COVID-19 restrictions were younger than those who continued using PrEP (mean: 42.44 vs. 45.24; P = 0.031). They were also less likely to have recently tested for both HIV and other sexually transmitted infections (STIs) than those who continued using PrEP.
Hill (2021)	Arkansas, Missouri, Oklahoma, USA	Quantitative	n/a	n = 80	Results revealed that a significantly greater proportion of male PrEP visits occurred during the first four months of the COVID-19 pandemic, compared to male PrEP visits during the same time period in the previous year (z = − 3.83, p < 0.001). This difference had a small effect size (d = 0.16). Monthly year-over-year comparisons of male PrEP visits suggest a significantly greater proportion of male visits were used for PrEP for every month during the onset of the pandemic with the exception of May (z = 0, p = 1.0, d = 0.01).
Hoagland (2021)	Brazil	Quantitative	n/a	n = 2375	PrEP teleconsultation was experienced by 21.5% of PrEP users (146/680) and 89.0% (130/146) reported feeling satisfied with these new procedures. High acceptability of PrEP teleconsultation was reported by 70%. In ordinal logistic model, having higher education was associated with high acceptability of PrEP tele- consultation (aOR:1.62; 95%CI: 1.07–2.45).
Hong (2022)	USA	Quantitative	Young MSM	n = 239	One-in-seven YSMM PrEP users discontinued use during the pandemic, and all those who discontinued PrEP reported a decrease in sexual activity. Among those who met Centers for Disease Control and Prevention criteria for PrEP (n = 104), 86.5% were not currently using PrEP.
Howarth (2021)	UK	Quantitative	MSM	n = 2018	Among all participants, 23.4% had ever used PrEP, 5.1% last used it just before lockdown and 15.4% last used PrEP since lockdown began. Among PrEP users (those who reported ever using PrEP), 21.7% had last used PrEP just before lockdown and 65.7% since lockdown began. Those who had last used PrEP since lockdown began were more likely to report one or more new partners since lockdown began (63.8% vs. 19.8%), whereas those who had last used PrEP just before lockdown were more likely to report no new partners since lockdown began (80.2% vs. 36.2%, p < 0.001).
Hyndman (2021)	UK	Quantitative	MSM	n = 814	75% of the MSM were PrEP users. PrEP users had a higher median number of sex partners and were more likely to have sex outside their household than non-PrEP users during lockdown. They were also more likely to engage in chemsex and were more likely to have accessed SHS (all p < 0 0.01), but there was no significant difference in reported STI diagnoses between the two groups.
Jongen (2021)	Amsterdam, Netherlands	Quantitative	MSM	n = 136	The proportion of days with PrEP use decreased from 74% before to 58% after March 15, 2020 (P < 0.001). After March 15, 2020, PrEP use during sex decreased with unknown casual partners (b = 20.36; 95% CI = 20.72 to 0.00) but not with steady partners and known casual partners.
MacCarthy (2020)	California, USA	Mixed methods	Latinx sexual minority men (LSMM) and transgender women (LTGW)	n = 52	More than one-third (35.6%) reported being on PrEP prior to COVID-19; among those previously taking PrEP, 66.7% have continued taking it and reported getting refills either via telemedicine appointments or office-based visits. One-quarter (25.0%) reported being either concerned or very concerned about their ability to access PrEP during the shelter at home order. The reasons for discontinuing PrEP related to a substantial decrease in the number of their sexual partners, so participants no longer felt like they needed to take it. A few participants drew parallels with HIV, saying they viewed COVID-19 as a highly infectious version of HIV and wanted to avoid it, and therefore continued taking PrEP.
Matambanadzo (2021)	Zimbabwe	Quantitative	female sex worker	n/a	Beginning May 2020, PrEP uptake increased monthly, peaking at an initiation rate of 51% (n = 1360) in September 2020. Unexpected rise in demand coincided with national commodity shortages between October and December 2020, resulting in restriction of new initiations with sites prioritizing refills.
Mistler (2021)	Connecticut, USA	Quantitative	Opioid-Dependent people who inject drugs	n = 110	One-fourth of the 32 participants who were taking PrEP before the onset of COVID-19 reported that they had trouble getting their PrEP prescription due to COVID-19, and some reported that they had difficulty getting the PrEP prescription filled at their pharmacy.
Muhula (2021)	Kibera, Kenya	Quantitative	n/a	n = 176	PrEP among discordant couples, the general populations, MSM, female sex workers and people with disabilities significantly increased by 24%
O’Byrne (2021)	Ottawa, Canada	Quantitative	n/a	n = 202	In a nurse-led PrEP clinic (PrEP-RN), PrEP linkage to care and follow-up occurred via established protocols. Among these 128 participants, 63.6% (n = 82/129) belonged to an HIV priority group and were offered a referral for HIV PrEP.
Pampati (2020)	Southern USA	Quantitative	MSM	n = 56	A fifth of participants discontinued or changed how often they take PrEP because of COVID-19. A quarter of the cohort documented challenges when attempting to access PrEP, HIV testing, or STD testing. Five participants (9%) reported discontinuing PrEP use. Several participants reported difficulties obtaining their PrEP medication (n = 8, 16%), and few participants noted switching to event-based dosing (n = 2, 4%).
Quirke (2021)	Ireland	Quantitative	n/a	n = 387	A small number of patients discontinued PrEP themselves during the lockdown period, as they were not at risk of HIV during this time and did not wish to take unnecessary medication. These patients have recommenced PrEP either themselves as they resumed sexual activity outside the home or since the reopening of the PrEP service. Others switched themselves from daily dosing to event-based dosing as required.
Rao (2021)	20 countries	Quantitative	MSM	n = 10,654	56% (5171/9173) reported perceived interruptions to PrEP. For PrEP, greater proportions reporting perceived interruptions were seen in Mexico and Turkey compared with all other countries.
Reyniers (2021)	Belgium	Quantitative	MSM	n = 694	Among those who used PrEP before the lockdown, 47.0% stopped using PrEP, 19.7% used event-driven PrEP and 33.3% used daily PrEP during the lockdown. Almost two-thirds of PrEP users had a PrEP care appointment in the weeks before the lockdown and a minority received follow-up elsewhere or online. Some PrEP users had concerns regarding their follow-up.
Ringshall (2021)	Brighton, UK	Quantitative	n/a	n = 109	A small but significant proportion of MSM using HIV-PrEP during the COVID-19 pandemic continued to see the same or increased number of non-steady sexual partners. These MSM were more likely to have engaged in chemsex and use STI-PrEP.
Roche (2021)	Kenya	Mixed method	n/a	n/a	From the pre- to post-period, average monthly initiations at intervention and control clinics increased by 6 and 2.3, respectively, and percent of expected follow-up visits occurring on time decreased by 18% and 26%, respectively; controlled interrupted time seris analysis of PrEP initiations (n = 1227) and follow-up visits (n = 2696) revealed no significant difference between intervention and control clinics in terms of trends in PrEP initiation and on-time returns (all p > 0.05).
Rogers (2021)	USA	Quantitative	Cis-gender MSM	n/a	During the Evolving Phase (compared to the Pre- COVID-19 Phase), there was a 6% [incidence rate ratio (IRR):0.94, 95% confidence interval (CI): 0.68–1.30, P = 0.72] reduction in the total number of PrEP visits, a 44% (IRR: 0.56, 95% CI: 0.21–1.50, P = 0.24) reduction in the number of initial PrEP visits, and a 0% (IRR: 1.00, 95% CI: 0.71–1.41, P = 0.99) decrease in the number of follow-up PrEP visits. During the COVID-19 Plateau Phase (com- pared to the Pre-COVID-19 Phase), there was a 16% (IRR: 0.84, 95% CI: 0.58–1.20, P = 0.34) reduction in total PrEP visits, a 49% (IRR: 0.51, 95% CI: 0.18–1.46, P = 0.21) reduction in initial PrEP visits, and a 12% (IRR: 0.88, 95% CI: 0.60–1.29, P = 0.51) reduction in follow-up PrEP visits. No significant decreases in PrEP visits were observed at any time point.
Rogers (2022)	USA	Quantitative	MSM	n = 177	PrEP users reported an average of 2.60 fewer sexual partners (95% CI − 4.04, − 1.40) during the pandemic compared to pre-COVID-19. Rates of depressive symptoms and alcohol use remained stable, and few patients reported substance use.
Saberi (2021)	San Francisco, USA	Quantitative	n/a	n = 267	Data indicate no reduction in PrEP use by patients in San Francisco Department of Public Health primary care clinics after the SARS-CoV-2 pandemic shelter-in-place restrictions.
Saberi (2022)	San Francisco, USA	Quantitative	n/a		Nearly 87.7% PrEP users noted being extremely to moderately satisfied with the ability to complete the laboratory tests without having to come into a clinic. Approximately 49.3% of participants chose this home-collection method as their first choice for providing laboratory samples.
Sousa (2020)	Brazil and Portugal	Quantitative	MSM	n = 92	Not living with the partner (aOR = 1.8; 95%CI: 1.2–2.6) and using PrEP (aOR = 2.6; 95%CI: 1.8–3.7) also substantially increased the odds of engaging in chemsex.
Stephenson (2021)	USA	Quantitative	MSM	n = 518	Current PrEP use was relatively high at 18%, with 9% reporting that COVID-19 had prevented them accessing their PrEP prescriptions.
Stephenson (2022)	USA	Quantitative	MSM	n = 280	Current PrEP use was relatively high at 21.8%, with 2.5% reporting that COVID-19 had prevented them accessing their PrEP prescriptions.
Torres (2021)	Brazil	Quantitative	MSM	n = 3486	A total of 68.5% (502/733) maintained daily oral PrEP during social distancing period, while 27.8% (204/733) stopped it completely, 1.5% (11/733) used ED-PrEP, and 2.2% (16/733), nonstandard PrEP regimens. Main reasons for stopping PrEP use were impediments to pick up PrEP refill at the health service (95/204; 46.6%) and sexual abstinence (81/204; 39.7%). Main reasons for continuing PrEP were fear of HIV infection (327/529; 61.8%), sex with casual partners (90/529; 17.0%), HIV-positive partner (63/529; 11.9%), and belief that PrEP protected against COVID-19 (49/529; 9.3%).
Traeger (2021)	Australia	Quantitative	n/a	n/a	PrEP prescriptions declined by an estimated 236 at the week following implementation of restrictions, representing an immediate 33.3% decline in prescriptions (P < 0.001). Between 1 April 2020 and 30 June 2020 (during-restrictions period), the average number of PrEP prescriptions per week was 543 (a 24.4% decline compared with the pre-restrictions period overall). There was a nonsignificant increase of 10.6 prescriptions per week during the restrictions period (P 1⁄4 0.178).
Richardson (2021)	UK	Quantitative	MSM	n = 448	Overall, 94/448 (21%,95% CI = 17–25) of MSM were using event-based (EBD)-PrEP. New starters were significantly more likely to use EBD-PrEP compared to existing PrEP users (34%.vs.13%, χ2 = 27.6, p < 0.00001). There were 33/38 clinicians who responded to the online survey. Clinicians felt equally confident at delivering daily PrEP as EBD-PrEP (Likert scores = 4.4/5 v 4.2/5, p = 0.2).
Xavier Hall (2022)	USA	Quantitative	SGM youth and young adults	n = 1142	This study was not sufficiently powered to observe a statistically significant association between social distancing and PrEP use. While it was not significant, those who reported no COVID-19 protective behaviors had the least amount of PrEP use compared to those with one or more social distancing behaviors.
Zapata (2022)	USA	Qualitative	Young MSM	n = 41	By PrEP use status, 22.0% of participants were currently taking PrEP, and 17.1% were former PrEP users. Three participants (7.3%) reported prior PEP use in their lifetime. PrEP services were also disrupted both in maintenance and initiation. Although several participants were on PrEP, they experienced barriers to attending appointments, receiving refills, and/or obtaining their quarterly testing.

MSM = men who have sex with men

TGW = transgender women

SGM = sexual and gender minority

### Reasons for Changing PrEP Uptake and Persistence

The most commonly reported reasons for changes in PrEP use and persistence are limited access to healthcare services, and changes in sexual behaviors and perceived risk of HIV. First, the pandemic and associated measures placed additional barriers to routine healthcare services. PrEP users said they were unable to get providers’ appointments or unable to communicate with their providers[44, 45]. Many also reported having difficulty refilling the prescription and getting their medication[21, 31, 32, 37, 55]. Qualitative results also further illustrated these barriers,

*“I couldn’t get PrEP filled because my appointment was cancelled because of the pandemic.”*[29]

Besides, the closure of the clinic and reduced hours of operation also negatively impacted PrEP persistence among PrEP users. Camp and Saberi found that the top PrEP challenge during the pandemic was laboratory testing[44], also suggested by Chan et al. [45]. On the other hand, studies also found that PrEP-related clinic visits had declined significantly. For example, in Belgium, PrEP users visited the clinic less frequently in 2020 than in 2019[24], and PrEP service had a 50% decline in visits in Croatia[62]. In South Africa, missed PrEP visits among pregnant women increased significantly during the nationwide lockdown[25]. In addition, one study in Providence, Rhode Island, discovered that based on their patient record in a PrEP program, there was a decrease in PrEP visits over time during three distinct phases of COVID-19. This decrease, however, was not statistically significant[35]. In a qualitative study among opioid-dependent individuals who inject drugs, a quarter of the participants said they had difficulty getting their PrEP prescription and had trouble refilling the medication at their pharmacy[38].

The other main reason for changes in PrEP use and persistence during COVID-19 was changes in sexual behaviors and perceived risk of HIV infection. Specifically, many studies found that those individuals who stopped using PrEP reported stopping sexual encounters or decreasing sexual activities[21, 44, 53, 55] and having a reduced number of casual sexual partners[39, 52, 53]. In a qualitative study, MacCarthy et al. discovered that PrEP discontinuation was related to a substantial decrease in the number of participants’ sexual partners, and these individuals, therefore, felt like they no longer needed to take PrEP[39]. Indeed, reduced sexual activities and the number of sexual partners also changed their perceived risk of HIV. In Ireland, the number of individuals seeking PrEP & PEP decreased dramatically during the pandemic. Some patients stopped PrEP themselves as they were not at risk of HIV and did not want to take unnecessary medication[63]. Another study also found that discontinuing PrEP during the lockdown was less likely to receive an HIV test recently[52].

Other factors associated with changes in PrEP use and persistence include younger age and financial vulnerability. For example, one study in Australia found that men who discontinued PrEP during the restrictions were significantly younger than those who continued using PrEP (mean age: 42.44 vs. 45.24, p = 0.0031). Rao et al. also found that younger women were more likely to discontinue PrEP by not returning compared to 25 years and older[27]. In a sample of Black MSM and transgender women in Chicago, the financial travel burden since the lockdown was significantly associated with perceived difficulty accessing PrEP[42].

Very few studies have identified the facilitators of PrEP use during the COVID-19 pandemic. One study in Brazil found that the main reasons for individuals continuing PrEP were fear of HIV infection, having sex with casual partners, having HIV-positive partners, and believing that PrEP protected against COVID-19[55]. A similar study also suggested nearly half of MSM in Brazil were taking PrEP to prevent COVID-19[54]. In South Africa, the most common reasons pregnant women start PrEP were preventing infant HIV and unknown or positive partner serostatus[25]. Chone et al. (2021) also found that using PrEP was associated with having chemsex during the pandemic in Portugal[58].

### Switch to on-demand PrEP

One emerging theme is that individuals switched oral-daily PrEP dosing to ‘event-driven’ or on-demand PrEP to continue the PrEP care during the pandemic[23, 37, 44, 50, 53, 63]. Among men who have sex with men who attended PrEP care at the Melbourne Sexual Health Centre, two studies found that 5% and 2% of the PrEP users switched to on-demand PrEP[23, 53]. Similar results were found in studies in the Southern U.S. and Ireland[37, 63]. One study further examined the barriers and facilitators of using the on-demand PrEP dosing option. According to Camp and Saberi[44], the most common reasons for switching from daily PrEP to on-demand PrEP were having sex less frequently and wanting to take less pills. The main barriers to using this dosing option include unexpected sexual behaviors that result in missing the first dose, difficulty remembering the dosing after the double dose, and lack of provider knowledge[44]. Their results also revealed high medication adherence based on the 2-1-1 component. Lastly, a study in the U.K. found that the uptake of on-demand PrEP had increased significantly since the pandemic began, and clinicians were confident to discuss this dosing option with the clients[50].

## Discussion

This review of 46 studies suggests that the global COVID-19 pandemic and associated measures have disrupted the continuity of PrEP care across various at-risk populations. Research has already suggested the negative impact of the COVID-19 pandemic on the HIV care and prevention continuum, including additional barriers to HIV and other STI testing, difficulties refilling antiretroviral therapy and seeing HIV care providers [4, 69]. Our results add to the evidence by suggesting disruptions in PrEP use and continuation. Most studies found that individuals in PrEP care stopped using PrEP or indicated difficulties in getting PrEP care. The most common reasons for stopping using PrEP were experiencing barriers to PrEP care services, reduced sexual behaviors, and lowered self-perceived risk of HIV. Only a few studies observed increased PrEP initiation and persistent PrEP use during the pandemic. However, these studies were conducted in a PrEP optimization or an integrated PrEP service program. Future research should investigate which implementation strategies these programs enacted had facilitated the positive changes, and such strategies should be continued and taken forward for improvement.

Of note, most of the included studies were cross-sectional and quantitative designs. Despite the fact that many studies asked their participants how PrEP use and persistence had changed since the pandemic, our knowledge of the longitudinal effect of the pandemic on PrEP care engagement is still limited. Future studies may consider utilizing longitudinal or cohort data to assess the pandemic’s long-term impact. Additional research using a qualitative approach may be a better way of more fully understanding the impact of the pandemic on PrEP use. Besides, a vast majority of the studies were conducted in high-income and developed countries and areas and only a handful of studies examined the changes in PrEP use and persistence in low- and middle-income countries and the Global South. Compared to high-income settings, PrEP scale up and implementation for HIV prevention have been slow, but a growing number of countries are adopting the WHO PrEP guidelines and recommendations[70]. In addition, researchers and policymakers must examine the changes in PrEP use and persistence in these areas to maintain the public health gains in global PrEP use and achieve greater PrEP equity, especially given the already existing barriers to PrEP implementation before the pandemic.

Despite most studies documenting the decrease in PrEP use, very few studies assessed PrEP eligibility and identified PrEP candidates during the pandemic. HIV risk should be evaluated in the context of behavioral risk exposure. For example, one study in the U.S. found that among those MSM who met the CDC’s PrEP criteria, 86.5% were not using PrEP[21]. To maximize the public health benefit of this biomedical intervention, assessments on PrEP eligibility should be conducted regularly to assess one’s need for PrEP care. During the pandemic, many PrEP clinics and organizations have limited operating hours and capacities for individual HIV risk assessment. Virtual services such as eHealth, telehealth, and technology-based intervention may mitigate the negative impacts of the COVID-19 pandemic on PrEP-related services[71, 72]. In addition, recent literature also suggests utilizing data science approaches to predict HIV risk behaviors and deliver individualized messages and interventions[73, 74]. Such novel approach may have the potential to maintain comprehensive HIV and PrEP prevention service delivery and address potential COVID-19 related service interruptions.

Consistent with pre-pandemic, young people continue to experience barriers to PrEP care. Despite being limited, several studies found that young MSM and young women were more likely to stop using PrEP during the pandemic. Globally, adolescents and young adults bear a disproportionately high burden of HIV infection [75] and continue to experience difficulty accessing and maintaining PrEP care[76, 77]. The ongoing pandemic could further exacerbate their already existing vulnerabilities. PrEP initiation for adolescents and young adults may have been delayed due to the pandemic, which could have long-term effects on PrEP use and persistence among this population. A recent study among adolescents aged 13–18 found that PrEP use was extremely low among sexually active YMSM, while risk behaviors such as condomless anal sex persist during the pandemic[78]. Public health programs and providers should prioritize this population for future HIV prevention efforts.

Lastly, there is a growing trend of switching to on-demand PrEP from daily dosing among PrEP users during the pandemic. On-demand PrEP or event-driven PrEP is an alternative dosing option for individuals with less frequent sexual encounters and fewer risk exposures[79]. This involves taking 2 pills 2–24 h before sex and 1 pill daily for the next 2 days and is therefore known as the “2-1-1” PrEP. It was first incorporated into European clinical guidelines in 2015 and endorsed by the World Health Organization in 2019[79, 80]. This change may be explained by the fact PrEP users only use PrEP when they are in need because of the shortage of medication supply and having difficulty refilling their pills due to the lockdown measures across the globe. It could also reflect the changes in one’s perceived risk of HIV and risk evaluation. Indeed, due to decreased frequency of sexual behaviors and the number of sexual partners, PrEP users may only intake the pills when needed. As the interest and demand for this dosing option are growing, it is critical for public health officials and providers to offer different options matching one’s needs and implement consistent and comprehensive knowledge and education about correct dosing and adherence for on-demand PrEP users. Areas and settings that bear a high HIV burden should work with their Public Health agencies to endorse on-demand PrEP dosing options and provide alternative PrEP modalities based on individual unique needs. Future studies should continue exploring novel PrEP modalities that will combat the barriers to traditional daily oral PrEP.

This review has several limitations. First, this review focused on reviewing the literature and summarizing the emerging evidence instead of providing a rigorous assessment of the quality of included studies. Secondly, only published studies in English were screened. It is possible that some studies in other languages were inadvertently missed. Lastly, due to the heterogeneity of the included studies, a meta-analysis was not performed to derive aggregate statistical evidence about the changes in PrEP use and persistence. Despite these limitations, this review highlighted the impact of the global COVID-19 pandemic on the engagement of PrEP care across different settings and populations and provided insights and evidence for future PrEP implementation programs and interventions.
